# Two different patterns for the premature ventricular complex induction of a long RP supraventricular tachycardia

**DOI:** 10.1016/j.hrcr.2022.02.003

**Published:** 2022-02-11

**Authors:** Xumiao Chen, Wei Tang, Chen Su, Xiaoyu Zhang, Lichun Wang

**Affiliations:** Department of Cardiology, The First Affiliated Hospital of Sun Yat-sen University, Key Laboratory on Assisted Circulation, Ministry of Health, Guangzhou, China

**Keywords:** Long RP, Supraventricular tachycardia, Fast–slow type of atrioventricular nodal reentrant, Premature ventricular complex, Diagnosis

**Key Teaching Points**

• The fast–slow type of atrioventricular nodal reentrant tachycardia (FS-AVNRT) is an infrequent type of AVNRT and is more likely to be induced by premature ventricular complex (PVC) or stimulation.
• When a supraventricular tachycardia could be terminated by PVC, the lack of a premature retrograde atrial activation could exclude the possibility of intra-atrial reentrant tachycardia.
• The V-A-A sequence without resetting of the A-A interval of tachycardia indicates the lack of stable ventriculoatrial relationship and could rule out permanent junctional reciprocating tachycardia.
• FS-AVNRT could be induced by PVCs through different patterns with/without a sinus P-QRS-T complex locating between the PVC and the first negative P wave of the tachycardia, which depended on whether the retrograde activation along the slow pathway conducted to the atrium before sinus activation.

## Introduction

Atypical fast–slow type of atrioventricular nodal reentrant tachycardia (FS-AVNRT) is a relatively infrequent type of AVNRT. It can be induced by ventricular stimulation more easily. However, the initiation of FS-AVNRT induced by ventricular activation is complex. Here we report a case of FS-AVNRT that was induced by premature ventricular complex (PVC) through 2 different patterns.

## Case report

**Figure 1:**
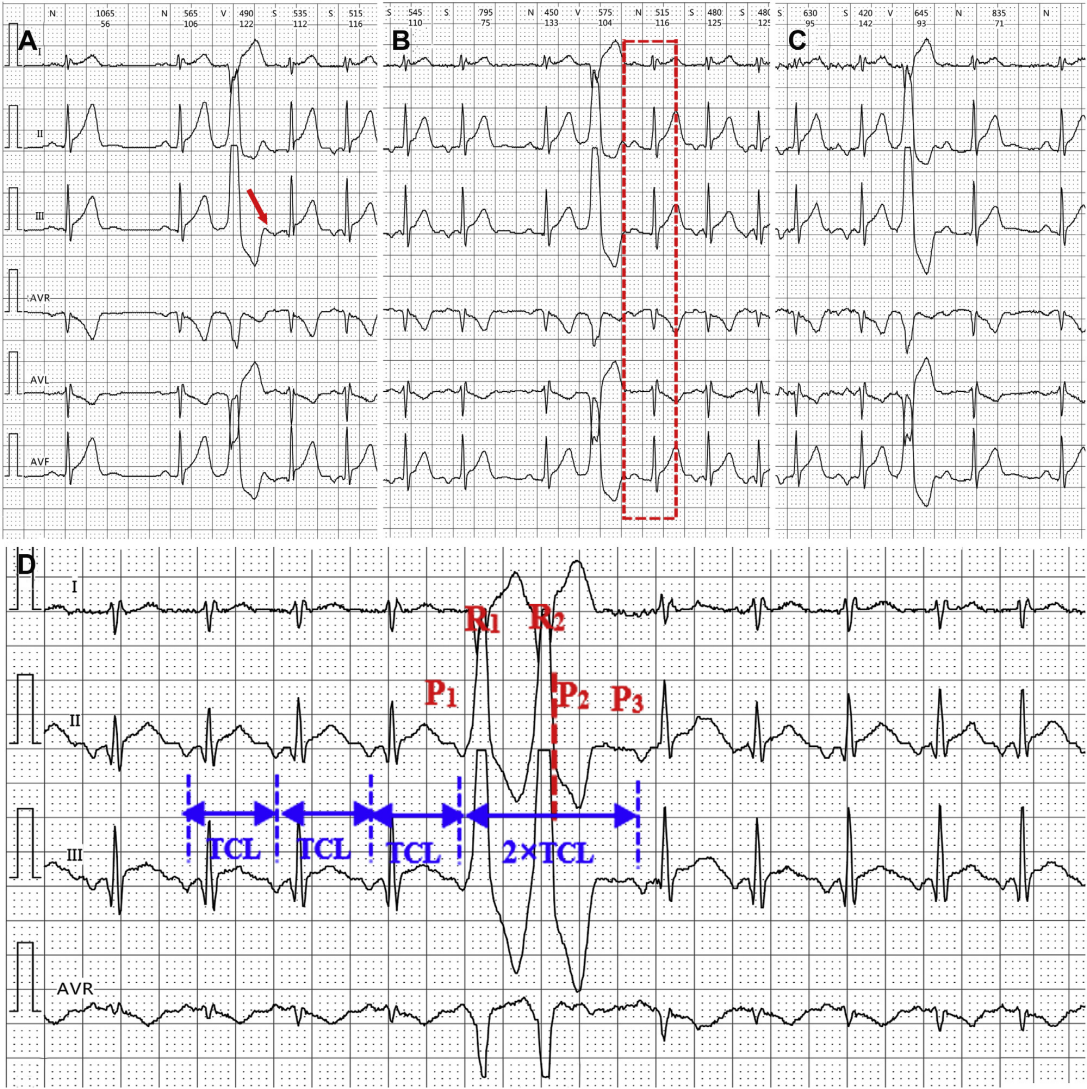
The electrocardiographic characteristics and diagnosis analysis of the long RP supraventricular tachycardia with negative P waves in inferior leads. **A:** The tachycardia was induced by premature ventricular contraction (PVC) with an inverted P wave following the PVC directly (*red arrow*). **B:** The tachycardia ended at the QRS wave automatically, and was reinduced by PVC with a sinus P-QRS-T wave (*red square frame*), which occurred between the PVC and the first inverted P wave of the tachycardia. **C:** The tachycardia was terminated by a single PVC without a premature retrograde atrial activation. This indicated atrial tachycardia was not possible. **D:** During the tachycardia, paired premature ventricular contractions (R_1_, R_2_) did not terminate the tachycardia. The interval of P_1_P_3_ was 2 times the tachycardia cycle length. So, a regular tachycardia P wave (P_2_) should closely follow the R_2_ (which could be seen indistinctly in the aVR lead). Therefore, R_2_ did not reset the P_3_, and no ventricular activation occurred between P_2_P_3_. This indicated the ventricle was not involved in the reentrant circuit.

A 33-year-old man with frequent episodes of palpitations for 3 months was referred to our hospital. Twenty-four-hour Holter recording (12 leads) showed frequent PVC (10,959 beats / 24 hours). Some PVCs could induce a long RP supraventricular tachycardia with negative P waves in the inferior leads (Figure 1). Interestingly, there were 2 different patterns for the PVC induction of tachycardia. One showed a negative P wave directly after PVCs preceding the first narrow QRS of the tachycardia (Figure 1A); the other presented with a sinus P-QRS-T complex between the PVCs and the first negative P wave of tachycardia (Figure 1B). The tachycardias often lasted from several seconds to 10 minutes, which could be terminated by PVCs or ended at QRS waveform spontaneously (Figure 1B and 1C). What is the diagnosis of the tachycardia? What are the mechanisms for the 2 different initiation patterns of the tachycardia?

## Diagnosis

### Electrocardiographic analysis

Given that the tachycardia could be induced or terminated by PVCs, the differential diagnosis of such a long RP supraventricular tachycardia with negative P waves in inferior leads would focus on (1) intra-atrial reentrant tachycardia, which broke out from the lower atrium (AT); (2) atypical FS-AVNRT; and (3) a slowly ventriculoatrial conducting accessory pathway–dependent permanent junctional reciprocating tachycardia (PJRT).

Among them, tachycardia induced and/or terminated by PVCs was least likely in AT but most possibly in PJRT because of the difference in the distance from the source of PVC to the reentrant circle of tachycardia. Moreover, when the tachycardia was terminated by a single PVC, the lack of a premature retrograde atrial activation further excluded the possibility of AT (Figure 1C).

The principal difference between FS-AVNRT and PJRT is whether atrium or ventricle is a part of the reentrant circuit. In PJRT, both atrium and ventricle are components of the reentrant circuit, and are activated sequentially. In Figure 1D, a pair of PVCs did not terminate the tachycardia. The P-P interval, which included the pair of PVCs (P_1_P_3_), was 2 times the P-P interval of tachycardia. This indicated a regular P wave (P_2_) following the end of the second PVC (R_2_), and the R_2_ did not reset the tachycardia (P_3_). Therefore, there was not a ventricular activation between 2 regular P waves (P_2_P_3_) of the tachycardia. This suggested the ventricle was not a part of the tachycardia reentrant circuit. Thus, PJRT was also ruled out in this case. Finally, FS-AVNRT was the possible 1 among the 3 diagnoses according to surface electrocardiogram.

### Electrophysiologic study

**Figure 2:**
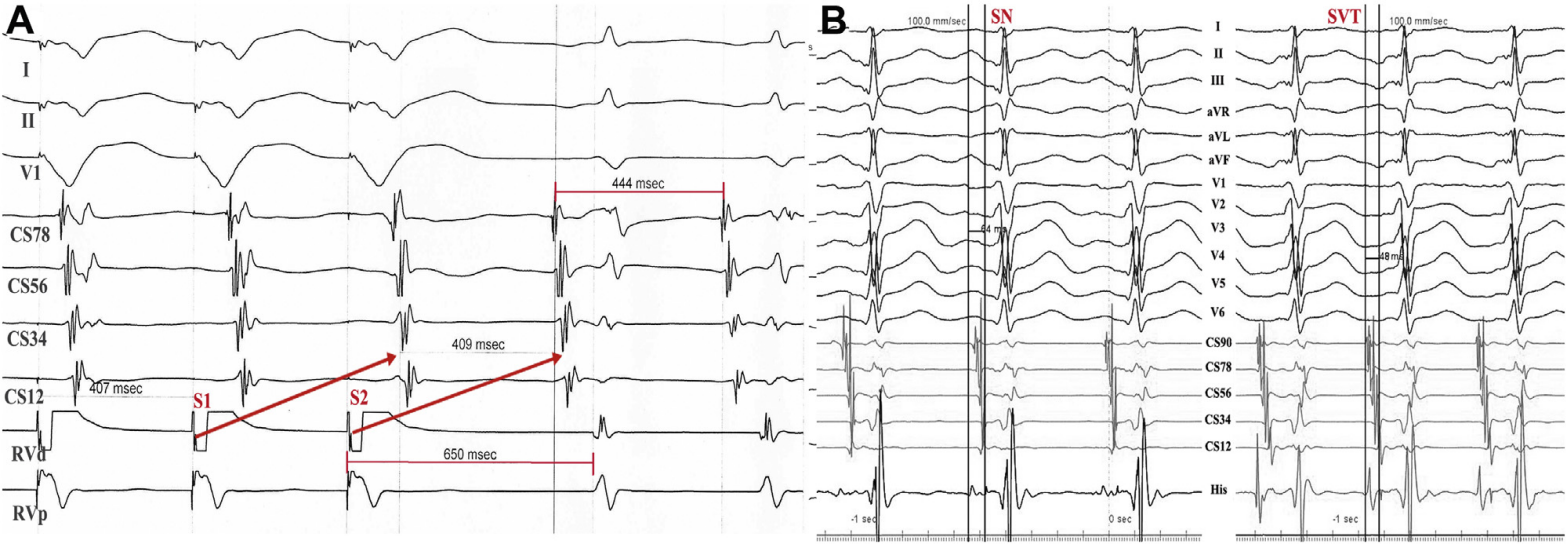
The electrophysiological characteristics of the tachycardia. **A:** There was a pseudo-V-A-A-V response to the ventricular overdriving pacing during the tachycardia because the retrograde conduction time was longer than the pacing cycle length (S1S2). **B:** The AH in sinus rhythm (64 ms) was longer than that in the tachycardia (48 ms). SN = sinus rhythm; SVT = supraventricular tachycardia.

Further intracardiac electrophysiologic study was performed. Although tachycardia could be induced by atrial programmed stimulation (S1S2) without an atrioventricular jump, it was easier to be induced by ventricular programmed stimulation. During tachycardia, a pseudo-VAAV restoring pattern produced by ventricular overdriving pacing ruled out IART. Meanwhile, the 206 ms difference of postpacing interval (650 ms) and the tachycardia cycle length (444 ms) did not favor the diagnosis of PJRT (Figure 2A). In addition, the shorter AH interval in tachycardia than sinus rhythm (Figure 2B) also suggested PJRT was most unlikely. Therefore, FS-AVNRT was diagnosed. Radiofrequency ablations of the slow pathway were performed along the tricuspid annulus immediately anterior to the ostium of the coronary sinus and successfully eliminated the tachycardia.

### Mechanism for the initiation of the tachycardia

As mentioned above, the tachycardia could be induced by PVC through 2 patterns. The mechanism for each pattern could be explained by the phenomenon of concealed conduction, which was determined by the difference of refractory periods in the fast and slow pathway.

**Figure 3:**
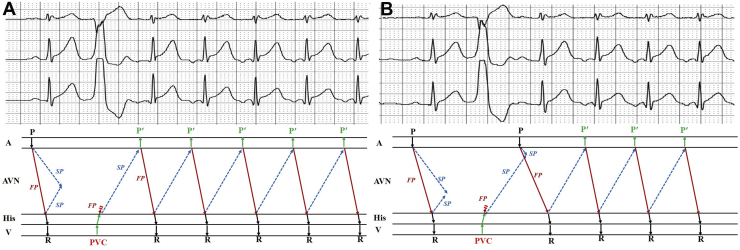
Depiction of the mechanisms of different initiations of the tachycardia. **A:** A premature ventricular contraction (PVC) induced the fast–slow atrioventricular nodal reentrant tachycardia (FS-AVNRT) with the inverted P wave directly following the PVC. The retrograde activation from the PVC was blocked in the fast pathway, but could conduct into the atrium through the slow pathway, which would produce an inverted “P” wave and initiate the tachycardia. **B:** PVC induced the FS-AVNRT with a sinus P-QRS-T complex, which occurred before the initiation of the tachycardia. The retrograde activation from the PVC was still blocked in the fast pathway and conducted slowly through the slow pathway. But before it came into the atrium, a sinus activation captured the atrium and conducted anterogradely through the fast and slow pathway. The anterograde activation would collide with the retrograde activation of the PVC in the slow pathway, and could only be conducted to the ventricle in the fast pathway. Meanwhile, the successive anterograde activation in the fast pathway could return back to the slow pathway and initiated the tachycardia. In this case, the PR of the sinus complex following the PVC was longer than the baseline PR; this indicated the anterograde conduction through the fast pathway after the PVC was slowed down because of the concealed conduction. AVN = atrioventricular node; FP = fast pathway; SP = slow pathway.

As a rule, the effective refractory period is longer in the fast pathway than in the slow pathway. In sinus beat, the anterograde activation along the fast pathway could return back to the slow pathway from its distal terminal and collide with the antegrade activation from its proximal terminal. Therefore, the time recovered from effective refractory period would be later in the fast pathway at both terminals of the atrioventricular node because of the nearly simultaneous activation in both fast and slow pathways. As a result, sometimes the retrograde activation from PVC could be blocked in the fast pathway but could conduct through the slow pathway. If this retrograde activation conducts to the atrium before sinus rhythm, an inverted P wave would appear and the activation could further antegrade pass through the fast pathway to initiate FS-AVNRT (Figure 3A). However, if sinus rhythm captured the atrium before the retrograde activation did, a sinus P wave would appear after the PVC, and its anterograde activation would collide with the retrograde activation from the PVC in the slow pathway. Meanwhile, along the fast pathway, the anterograde activation could conduct to activate the ventricle (a sinus P-QRS-T complex) and might return back to the slow pathway to initiate an FS-AVNRT (Figure 3B).

## Discussion

FS-AVNRT is relatively infrequent; it can be seen in 6.4% of patients with AVNRT.^1^ Unlike typical slow-fast AVNRT, it is assumed that the antegrade limb of FS-AVNRT is the fast atrioventricular nodal pathway, while 1 or more slow atrioventricular nodal pathways are considered as the retrograde limb. Although FS-AVNRT can be induced by both atrial and ventricular stimulation, it is more effective for ventricular stimulation, and in some patients the FS-AVNRT is induced only by ventricular stimulation.^2^ In this case, the fact that the tachycardia could be repeatedly induced by spontaneous PVCs or programmed ventricular stimulation further supports this point.

FS-AVNRT should be differentiated from IART and PJRT. In this case, the manifestation that PVC terminated the tachycardia without a premature retrograde atrial activation and a pseudo-V-A-A-V response to ventricular overdrive pacing ruled out the diagnosis of atrial tachycardia.^3^ In addition, the dissociation of atrium and ventricle in tachycardia (Figure 1D) excluded PJRT.

The interesting thing in this case is that we found the tachycardia was induced by single or paired PVCs through 2 different patterns. One showed a negative P wave directly after PVC, while the other presented a sinus P-QRS-T complex between the PVCs and the first negative P wave of tachycardia. A similar case to the latter was reported by Wellens^4^; however, in his case a PJRT was diagnosed.^4^ In our case, we proposed hypothetical models of the FS-AVNRT tachycardia circuits induced by PVC. Just as Figure 3 showed, the manifested pattern for the initiation of the tachycardia depended on whether the retrograde activation along the slow pathway conducted to the atrium before sinus activation.

## Conclusion

FS-AVNRT is an infrequent type of AVNRT and is more likely to be induced by PVC or ventricular stimulation. Our case indicated FS-AVNRT could be induced by PVC through different patterns with/without a sinus P-QRS-T complex locating between the PVC and the first negative P wave of the tachycardia.
